# A novel concatenate feature fusion RCNN architecture for sEMG-based hand gesture recognition

**DOI:** 10.1371/journal.pone.0262810

**Published:** 2022-01-20

**Authors:** Pufan Xu, Fei Li, Haipeng Wang

**Affiliations:** 1 School of Electronic Science and Engineering, Southeast University, Nanjing, Jiangsu, China; 2 Institute of RF- & OE-ICs, Southeast University, Nanjing, Jiangsu, China; 3 School of Electronic and Information Engineering, Sanjiang University, Nanjing, Jiangsu, China; Politechnika Slaska, POLAND

## Abstract

Hand gesture recognition tasks based on surface electromyography (sEMG) are vital in human-computer interaction, speech detection, robot control, and rehabilitation applications. However, existing models, whether traditional machine learnings (ML) or other state-of-the-arts, are limited in the number of movements. Targeting a large number of gesture classes, more data features such as temporal information should be persisted as much as possible. In the field of sEMG-based recognitions, the recurrent convolutional neural network (RCNN) is an advanced method due to the sequential characteristic of sEMG signals. However, the invariance of the pooling layer damages important temporal information. In the all convolutional neural network (ACNN), because of the feature-mixing convolution operation, a same output can be received from completely different inputs. This paper proposes a concatenate feature fusion (CFF) strategy and a novel concatenate feature fusion recurrent convolutional neural network (CFF-RCNN). In CFF-RCNN, a max-pooling layer and a 2-stride convolutional layer are concatenated together to replace the conventional simple dimensionality reduction layer. The featurewise pooling operation serves as a signal amplitude detector without using any parameter. The feature-mixing convolution operation calculates the contextual information. Complete evaluations are made on both the accuracy and convergence speed of the CFF-RCNN. Experiments are conducted using three sEMG benchmark databases named DB1, DB2 and DB4 from the NinaPro database. With more than 50 gestures, the classification accuracies of the CFF-RCNN are 88.87% on DB1, 99.51% on DB2, and 99.29% on DB4. These accuracies are the highest compared with reported accuracies of machine learnings and other state-of-the-art methods. To achieve accuracies of 86%, 99% and 98% for the RCNN, the training time are 2353.686 s, 816.173 s and 731.771 s, respectively. However, for the CFF-RCNN to reach the same accuracies, it needs only 1727.415 s, 542.245 s and 576.734 s, corresponding to a reduction of 26.61%, 33.56% and 21.19% in training time. We concluded that the CFF-RCNN is an improved method when classifying a large number of hand gestures. The CFF strategy significantly improved model performance with higher accuracy and faster convergence as compared to traditional RCNN.

## 1 Introduction

Electromyography (EMG) measures bioelectric currents produced by motor units during muscle contraction [1]. Surface EMG (sEMG) detects the sum of the motor unit action potential (MUAP) over the skin [2]. Due to its noninvasive and low-cost characteristics [3], sEMG-based hand gesture recognition systems are widely used in human-computer interaction [4], speech detection [5], robot control [6], and rehabilitation studies, like prosthesis operation [7–9] and stroke rehabilitation [10].

A traditional method for sEMG-based recognition is machine learning, which in general is not inherently efficient or scalable enough to handle massive datasets [11]. For simple pattern recognition (PR) based on sEMG signals [12–15], methods including linear discriminate analysis (LDA), k-nearest neighbor (KNN), principal component analysis (PCA), and artificial neural network (ANN) are usually chosen. Because of the stochastic nature of biological signals [16], signal preprocessing and feature extraction are necessary steps when applying these algorithms [17]. Data preprocessing, such as filtering, may result in the loss of valid information [18]. Feature extraction in machine learning is time-consuming and error-prone as it requires specialization, which significantly increases chances of reduced classification accuracy [19].

Deep learning methods are capable of handling large data calculation and perform auto-feature extraction in PR tasks. A model based on You Only Look Once (YOLO) v3 and DarkNet-53 convolutional neural networks (CNN) was built for image-based gesture recognition without additional data processing [20]. In CNN models, feature extraction is replaced by interconnected convolutions in the hidden layers, improving classification accuracies [21–23].

To further consider temporal information and process sequential data, state-of-the-art deep learning structures in time-series classification are welcomed in biosignal processing. A 50-layer CNN based on residual networks (ResNet) was built to classify 7 different gestures based on EMG [3]. Three architectures were designed for electrocardiography (ECG) classification, which were InceptionTime, ResNet and XResNet [24]. A novel spatial–temporal transformer network was proposed in reference [25] to solve skeleton-based human activity recognition tasks. RNNs also achieved high accuracies in speech recognition, signal detection, and video classification [26–28]. Combining the advantages of CNNs and RNNs, RCNNs have shown good performances in object detection, video classification, and emotion recognition [29–31]. Their merits when dealing with sEMG signals have also been proven both in discrete-motion classification and continuous-motion estimation [32, 33].

### However, existing hand gesture recognizers mostly recognize only a few movements

Classifying an average of more than 50 gesture classes led to a lower than 2% average chance level [34, 35]. By simply reducing the number of gestures, the classification accuracy exceeded 90% [36]. An LSTM-CNN (LCNN) achieved 98.14% of accuracy when classifying 5 hand gestures using a proposed MyoDataset, but only 71.66% using NinaPro DB5 Exercise A (12 movements) and 61.4% using NinaPro DB5 Exercise B (17 movements) [37]. This is likely due to the conventional RCNN structure, where the CNN part includes a 1-stride convolutional layer and a pooling layer, with the pooling layer playing a role in dimensionality reduction [38]. Max-pooling tosses information about the precise position of the entity within the region [39], resulting in significant degradation of sequential features [40]. Springenberg describes ACNN structure to replace the pooling layer with a normal convolution having a stride larger than 1 [38], expecting to gain more learnings of contextual information from the receptive field of the kernel. However, except the ability of information extraction, ACNN also has an information confusion characteristic. Because with the feature-mixing convolution operation, a same output can be received from completely different inputs. More details are explained in the Methods section. Therefore, it is important to develop an innovative method of dimensionality reduction to allow more classes in hand-gesture recognition tasks.

The main contributions of this work are twofold:

1. This paper introduces a new dimensionality reduction strategy, which is named the concatenate feature fusion (CFF) strategy. Under the CFF strategy, a max-pooling layer and a convolutional layer with a stride of 2 are concatenated together to replace the single max-pooling in CNN or the 2-stride convolution with in ACNN. The featurewise nature of the pooling operation maintains signal intensity, while the feature-mixing of the convolution operation obtains temporal information from the context.

2. This paper proposes a concatenate feature fusion recurrent convolutional neural network (CFF-RCNN) structure based on a traditional RCNN network, which consists of a 4-layer CNN and a long short-term memory network (LSTM). Classifications of more than 50 gestures are achieved with high accuracies and fast convergence using the CFF-RCNN structure. The classification accuracies of the CFF-RCNN on DB1, DB2 and DB4 three databases in the NinaPro are 88.87%, 99.51% and 99.29%, which are higher than the machine learnings, RCNN, and other state-of-the-art methods. When analyzing the convergence rate of the RCNN and CFF-RCNN, training times to reach the same classification accuracy are compared. To achieve accuracies of 86%, 99% and 98%, the training times are 2353.686 s, 816.173 s and 731.771 s for RCNN, while 1727.415 s, 542.245 s and 576.734 s for CFF-RCNN, corresponding to a reduction of 26.61%, 33.56% and 21.19%.

The organization of this paper is as follows. Section 2 discusses the novel CFF technique and the basic construction of CFF-RCNN. In Section 3, we explain the experiment methods to test the performance, accuracy and efficiency, and tolerance of CFF-RCNN. Results are presented and analyzed in Section 4. Finally, limitations are discussed and conclusions are drawn in Section 5.

## 2 Methods

### 2.1 The theory of CFF strategy

The pooling operation can be viewed as a featurewise convolutional layer with a p-norm activation function [38]. It is a significant component in CNNs for three main reasons: 1) neighboring pooling units take input from patches that are shifted by more than one row or column, making the representation of movements and distortions more invariant; 2) the spatial dimensionality reduction reduces the computational cost for the subsequent conventional layers; and 3) the featurewise nature could make optimization easier than the feature-mixing nature of the convolution [38, 41].

The first reason plays an important role in image recognition tasks where the locations of objects have less important features [42]. Therefore, the invariance of the pooling allows representations to change very little when elements in the previous layer vary with position and appearance [41]. However, the invariance characteristic has been recently questioned. By extracting some entity from the context, the clutter and noise are removed, but the information of the background is also damaged [43]. Additionally, contextual information, corresponding to the temporal information when inputting a sequence signal, is extremely important during the recognition process. Therefore, the pooling layer may result in heavy losses of crucial data features.

**Fig 1:**
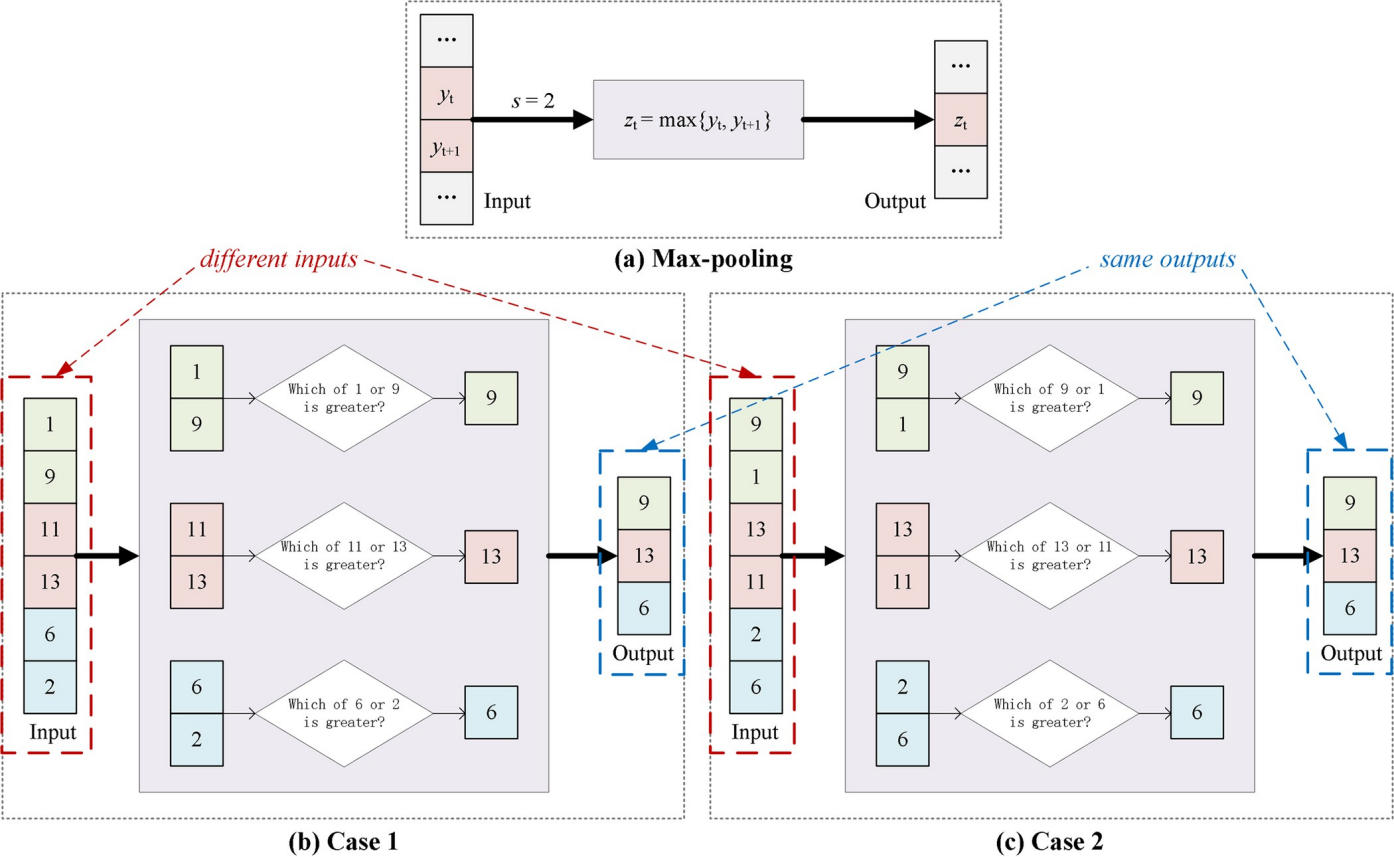
The procedure of the max-pooling layer. (a) The featurewise operation will result in information losses, where *y*_*t*_ is the *t*-th element of the input, *z*_*t*_ is the output, *s*_*p*_ is the pooling size and *s*_*s*_ is the stride (*s*_*p*_ = *s*_*s*_ = *s =* 2). (b, c) Examples of the invariance characteristic. Case 1 and 2 are different in the order. The output of the max-pooling layer cannot reflect small input changes, losing important sequential information.

To obtain a more quantized understanding of the information loss, the featurewise operation of 1D max-pooling can be described as follows:

$$z_{t}=\max\{y_{t},y_{t+1},\cdots ,y_{t+s-1}\}$$ (1)

where *y*_*t*_ is the *t*-th element of a row in the input map, *z*_*t*_ is the output map, *s*_*p*_ is the pooling size and *s*_*s*_ is the stride. Usually, the pooling size and stride are equal (*s*_*p*_ = *s*_*s*_ = *s*). Clearly, the max-pooling will throw away *s*-1 elements for each step, which is also shown in Fig 1A. Additionally, in Fig 1B (Case 1) and Fig 1C (Case 2), when only changing the order of the inputs, the output *z*_*t*_ remains unchanged. This is the so-called invariance characteristic, which will result in a serious loss in sequential information.

We symbolize the input data map of the *i*-th 1D convolutional layer as *X*_*i-1*_ and the output map as *X*_*i*_. The final output of the *n*-th convolutional layer is *Y* = *X*_*n*,_ and there are *n* layers of 1-stride convolution in total. The kernel sizes are unified to *k* and there is no padding. Considering a single convolution step as well, the output element *x*_*i*,*t*_ can be calculated to *k* input elements by a convolutional function *f*_*i*_:

$$x_{i,t}=f_{i}(x_{i-1,t},x_{i-1,t+1},\cdots ,x_{i-1,t+k-1})$$ (2)

$$\begin{array}{c} y_{t}=f_{n}\left(x_{n-1,t},x_{n-1,t+1},\cdots ,x_{n-1,t+k-1}\right)\\ =f_{n}\left(f_{n-1}(x_{n-2,t},x_{n-2,t+1},\cdots ,x_{n-2,t+k-1}),\cdots ,f_{n-1}(x_{n-2,t+k-1},x_{n-2,t+k},\cdots ,x_{n-2,t+2\cdot (k-1)})\right)\\ =F\left(x_{0,t},x_{0,t+1},\cdots ,x_{0,t+n(k-1)}\right) \end{array}$$ (3)

Then, *y*_*t*_ is related to *n*(*k*-1)+1 input elements. Therefore, after a max-pooling layer with a size of *s*, there will be a total loss of *s*-1 pieces of sequential information between *n*(*k*-1)+1 input data, which violently affects the performances of the following networks.

To achieve dimensionality reduction without the use of pooling, an ACNN structure was introduced by Springenberg, with the pooling layer replaced by a normal convolution with a stride larger than 1 [38]. It is worth noting that this improvement was based on an important assumption that only the second reason mentioned above, which is dimensionality reduction, was crucial for achieving good CNN performance. However, in this paper, the existence of max-pooling is questioned mainly because it abandons crucial sequential information. By simply removing the max-pooling and adding the convolution with stride larger than 1, the receptive field of the kernel may be helpful for learning some contextual information, but the results are probably not that good, even causing misleading guidance. To explain this, let us consider a convolution with a size of *k* and stride of *s*. The single-step operation (without bias and before activating) can be described as follows:

$$y_{t}=\sum_{j=1}^{k}\left(c_{j}\times x_{s(t-1)+j}\right)$$ (4)

where *x* is the input feature, *y* is the output feature, and *c* is the element in the convolutional kernel. Therefore, *y* is a summation of *k* input features, and the weights are learned during the training procedure. With different inputs and weights, chances are that the output feature *y* may be the same:

$$y_{t}=\sum_{j=1}^{k}\left(c_{j}\times x_{s(t-1)+j}\right)=\sum_{j=1}^{k}\left(c_{j}^{\prime }\times x_{s(t-1)+j}^{\prime }\right)$$ (5)

**Fig 2:**
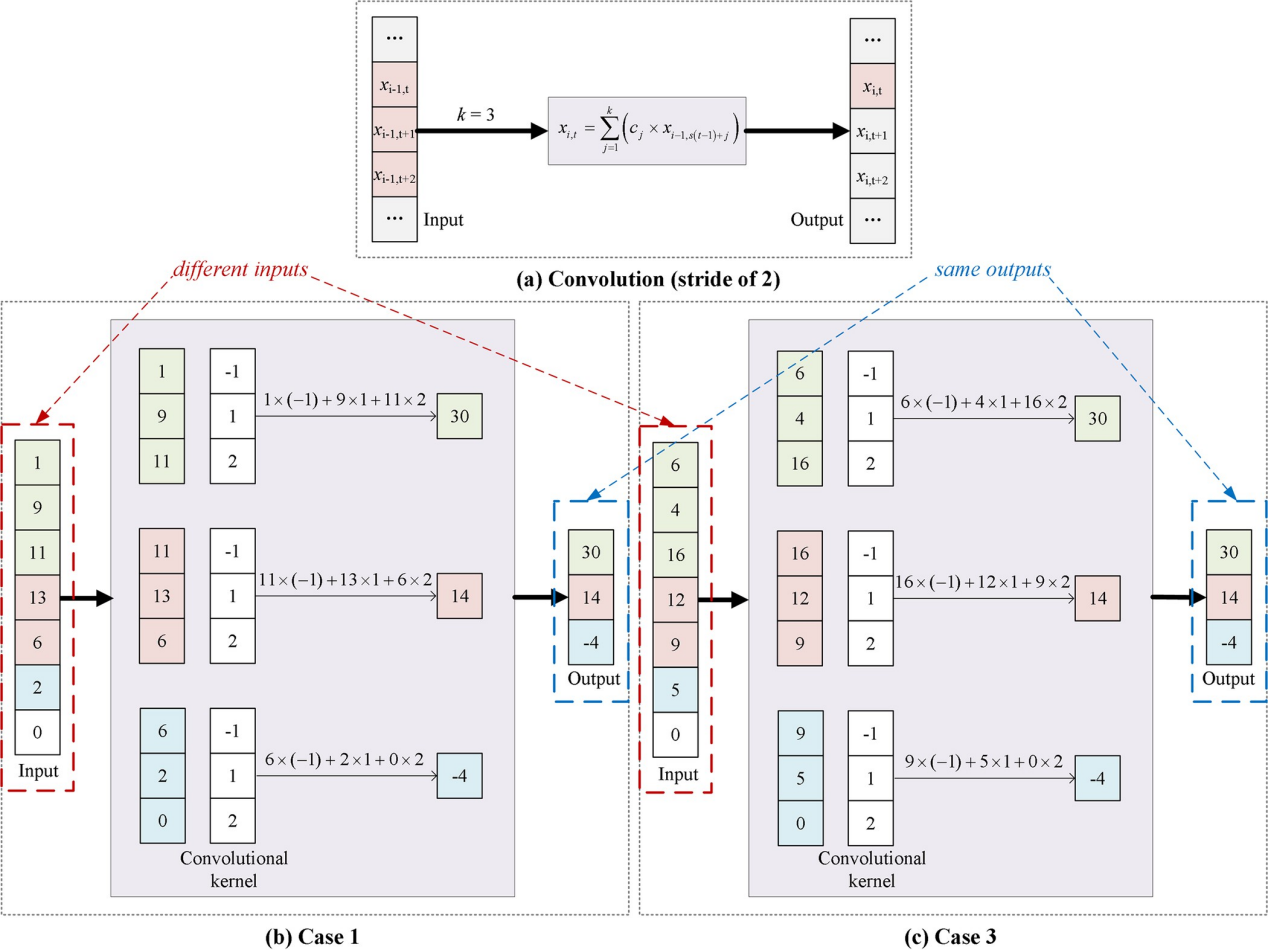
The procedure of the convolutional layer. (a) The feature-mixing operation of the convolution. *x* is the input feature, *y* is the output feature, and *c* is the element in the convolutional kernel. The kernel size is *k* (*k* = 3) and stride is *s*. (b, c) Examples of the information confusion caused by the feature mixing operation of the convolution. With the same convolutional kernels, same outputs can be received even though the input data are completely different in Case 1 and 3.

An example is shown in Fig 2. Fig 2A shows that when the kernel size *k* is 3, each output element *x*_*i*,*t*_ in the i-th layer is related to 3 inputs, *x*_*i-*1,*t*_, *x*_*i-*1,*t*+1_, *x*_*i-*1,*t*+2_, in the (i-1)-th layer. In Fig 2B (Case 1) and Fig 2C (Case 3), both the convolutional kernels are set at (-1, 1, 2). Although the input data are completely different, same outputs are received. To take a more extreme example, assuming that all the input data *x’* are unified to a constant *a* and $c_{j}^{\prime }=c_{j}\times x_{s\times (t-1)+j}/a$, the same output feature *y* is obtained. Therefore, *y* represents a time-alternating signal and a constant signal at the same time, resulting in confusion. The ability to extract the sequential information of the *s*-stride convolution mainly depends on the training effect, which is sensitive to the input data and increases computing loads.

**Fig 3:**
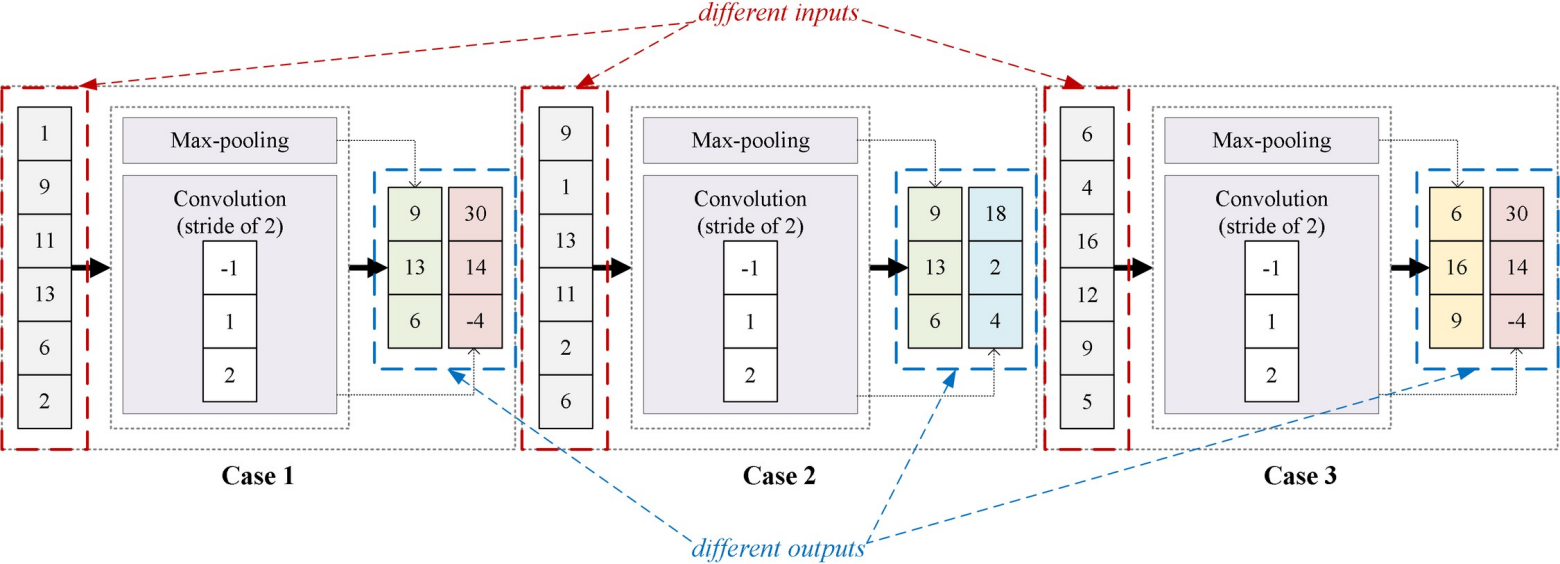
The procedure of the CFF strategy. Both the max-pooling and the 2-stride convolution are applied to the inputs. After doing so, distinguishable features are obtained to represent different sequential data.

The dual characteristics, which are information extraction and information confusion, are related to the feature mixing operation of the convolution. Although max-pooling fails to determine contextual features, it is an excellent featurewise amplitude detector without any training parameter. Knowing the most representative intensity of a sequence, the context information can be retained as much as possible. Therefore, a new dimensionality reduction strategy, which is called the CFF strategy, is proposed in this paper. The concatenate of a max-pooling layer and a convolutional layer with the stride of 2 is used to replace the traditional pooling layer in CNN, or the single 2-stride convolution in ACNN. As illustrated in Fig 3, both the max-pooling and the 2-stride convolution are applied to the inputs. Different outputs are received from Case 1 and 2 (which have the same result when only using max-pooling), and Case 1 and 3 (which have the same result when only using 2-stride convolution). After doing so, distinguishable features are obtained to represent different sequential data. By concatenating a max-pooling layer with a 2-stride convolutional layer, both featurewise and feature-mixing calculations are performed on the same data segment, extracting sequential information efficiently.

### 2.2 The architecture of the CFF-RCNN model

**Fig 4:**
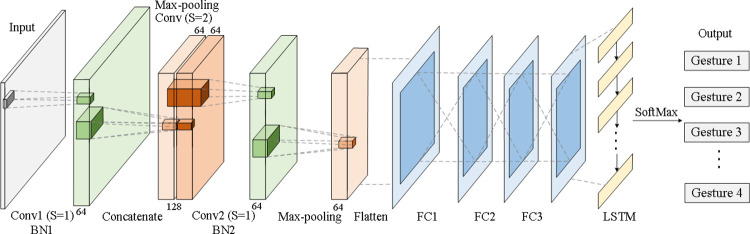
The structure of the CFF-RCNN.

Because of the reasons mentioned above, in this paper, a CFF-RCNN is constructed. The dimensionality reduction part uses the CFF strategy, operating as a feature fusion part at the same time (a dimension reducer and feature fusor). For the RCNN architecture, the CNN plays a role as a feature extractor and the RNN as a sequence recognizer. The detailed structure is shown in Fig 4.

Initially, the sEMG signals recorded from the electrodes of *C* channels are split into segments using an *s*-length sliding window with a *p*-length overlap. Each subsegment is denoted as *X*_*t*_*’*, where *X*_*t*_*’* contains *N* steps of data with *C* channels. Therefore, the size of *X*_*t*_*’* will be (1×*L*), where *L* = *N*×*C* (*L* is the length). Subsequently, *X*_*t*_*’* is reshaped into a 3D frame *X*_*t*_ with size (*W*×*H*×*C*), where *W*×*H* = *N* (*W* is the width and *H* is the height). The input data of the network are *X* = {*X*_*1*_, *X*_*2*_, …, *X*_*T*_}, where *T* is the total number of samples. A time-distributed CNN structure is applied as the feature extractor, where all the kernel sizes are 3 for the convolutions. The first layer is a 64 convolutional layer with a stride of 1. Then, the output feature maps are transmitted to both the max-pooling layer and the 2-stride convolutional layer in parallel. The pooling size is 2, and the number of filters is 64. After that, the output map of the convolutional layer is concatenated immediately after that of the pooling layer, forming a feature map with 128 channels. Furthermore, to extract detailed features, another 1-stride convolutional layer with 64 filters is placed after the concatenate operation. In addition, a simple max-pooling layer is used for dimensionality reduction. The last three layers of the feature extractor are fully connected layers, with 512, 512, and 128 hidden units. The rectified linear unit (ReLU) is chosen as the activation function, and batch normalization is used for all the 1-stride convolutional layers mentioned above. For the sequence recognizer, an RNN will be connected with the outputs of the CNN feature extractor. Each LSTM unit has 512 nodes and a dropout rate of 0.5, followed by a fully connected layer and a softmax layer as the classifier. A comparison of structures between the RCNN and the CFF-RCNN is presented in Table 1.

**Table 1:** The structure comparison between the RCNN and CFF-RCNN.

RCNN	CFF-RCNN	
Conv. 64 3	Conv. 64 3	
Batch Normalization	Batch Normalization	
Max-pooling 2	Max-pooling 2	Conv. 64 3/ S = 2
	Concatenate	
Conv. 64 3	Conv. 64 3	
Batch Normalization	Batch Normalization	
Max-pooling 2	Max-pooling 2	
Flatten	Flatten	
Fully connected 512	Fully connected 512	
Fully connected 512	Fully connected 512	
Fully connected 128	Fully connected 128	
LSTM 512	LSTM 512	
SoftMax	SoftMax	

## 3 Experiments

### 3.1 Experiment setups

Data acquisition: Performance of the novel CFF-RCNN model is evaluated using the first, second and fourth subdatasets of the NinaPro database [34, 44], denoted as DB1, DB2, and DB4. Detailed information on the databases is shown in Table 2. DB1 contains a total of 53 gestures (rest included) from 27 subjects, including 12 basic movements of the fingers (Exercise A), 8 isometric and isotonic hand configurations and 9 basic movements of the wrist (Exercise B), and 23 grasping and functional movements (Exercise C). It was recorded by 10 electrodes with a sampling rate of 100 Hz. DB2 collects 50 movements from 40 subjects, including 8 isometric and isotonic hand configurations and 9 basic movements of the wrist (Exercise B), 23 grasping and functional movements (Exercise C), and 9 force patterns (Exercise D). DB4 has 53 movements from 10 subjects, including 12 basic movements of the fingers (Exercise A), 8 isometric and isotonic hand configurations and 9 basic movements of the wrist (Exercise B), and 23 grasping and functional movements (Exercise C). Twelve electrodes were used to record the sEMG signals at a sampling rate of 2 kHz for both DB2 and DB4.

**Table 2:** Details of the three sEMG benchmark databases.

Database	Gestures	Channels	Subjects	Sampling rate (Hz)	Window length (ms)
**DB1**	53 (Exercise A, B & C)	10	27	100	250
**DB2**	50 (Exercise B, C & D)	12	40	2000	250
**DB4**	53 (Exercise A, B & C)	12	10	2000	250

Data preprocessing: Several signal processing steps including synchronization, relabeling and filtering were performed in the Ninapro databases [34]. All the data streams were synchronized to the high-resolution timestamps (100 Hz for DB1, 2 kHz for DB2 and DB4) using linear interpolation. The resulting erroneous movement labels have been corrected by applying the generalized likelihood ratio algorithm and the Lidierth threshold based algorithm. The Delsys electrodes for DB2 and Cometa sensors for DB4 are not shielded against power line interferences. Therefore, prior to synchronization, these signals were cleaned from 50 Hz (and harmonics) power-line interference using a Hampel filter. Standardization and normalization procedures were applied, and labels were encoded using one-hot encoder.

Data segmentation: As described in Section 2.2, the pre-processed data are segmented using the sliding window method. The raw subsegment *X*_*t*_*’* contains *N* steps of data with *C* channels and is reshaped into a 3D frame *X*_*t*_ with size (*W*×*H*×*C*), where *W*×*H* = *N* (*W* is the width and *H* is the height). In this paper, a 250 ms window with a 200 ms overlap is used for segmentation. For DB2 and DB4, *N* is set to 500 by simply multiplying the window length and the sampling frequency, *W* and *H*, which are set to 5 and 100, respectfully. This forms the input data with size (5×100×12). For DB1, *N* is 25. In addition, owing to the downsampling of DB1, the input data are reshaped into (5×25×2) to obtain larger data maps.

Training details: For the proposed CFF-RCNN and compared RCNN structures, stochastic gradient descent with momentum (SGDM) is chosen as the optimizer with a momentum of 0.95. The initial learning rate is set at 0.002, and the weight decay is set at 0.0005. The loss function is cross-entropy loss: $L\mathrm{oss}=-[y\log\hat{y}+(1-y)\log(1-\hat{y})]$, where *y* is the original label, and $\hat{y}$ is the result of the classification. The minibatch size is 20. The training epochs are set to 30 for DB2 and DB4 and 50 for DB1 due to the smaller map size. A 5-fold cross validation is used for model comparison. The networks operated on a workstation with an Intel Xeon E5-4210v2 central processing unit@ 2.2 GHz, NVIDIA GeForce GTX 2080Ti, and 128 GB access memory. The TensorFlow and Keras deep learning frameworks are used to design and train the networks.

### 3.2 Performance evaluation

To test the performance of the RCNN and CFF-RCNN, comparisons are made with traditional machine learning methods PCA, LDA and ANN and other state-of-the-art methods using the databases mentioned above.

We select four commonly used features, mean absolute value (MAV), zero crossings (ZS), slope sign changes (SSC), and waveform length (WL) to examine feature extraction performance. These features are calculated from each sEMG channel in a 250 ms sliding window with a 200 ms overlap in all methods tested. Both the PCA and LDA are used to reduce the dimensionality and analyze components, together with a KNN operating as a classifier. For ANN, three fully connected layers are built with the activation of ReLU, and the numbers of nodes are 100, 200, and 160. A dropout layer is added with a rate of 0.5, and softmax is used for classification. The ANN shares the same training optimizer and loss function with CFF-RCNN and RCNN, where the batch size is set to 32 and the number of epochs is 100.

We also compared with models from other works on the same benchmark dataset with all gestures. Traditional machine learnings tested include support vector machines (named LS-SVM) [45], random forests (RF) [34, 44] and LDA [45]. Other state-of-the-art approaches are AtzoriNet [34], ZhaiNet [46], transfer learning multi-scale kernel convolutional neural network (TL-MKCNN) [47], few-shot learning- hand gesture recognition (FS-HGR) [48], RNN with weight loss [49], a long short-term memory network with a multi-layer perceptron (LSTM+MLP) [50], CNN-LSTM [51], and attention-based hybrid CNN-RNN with feature-signal-image1 [32].

### 3.3 Comparison of the RCNN and CFF-RCNN

Experiments are designed to compare **accuracy**, **efficiency**, and **tolerance** of the RCNN and CFF-RCNN.

#### 3.3.1 Experiment 1: Accuracy and efficiency test

The first experiment is set to compare prediction accuracies, total training times and training times at different training epochs. To present the training process, the epoch number is changed from 30 to 10 with a step of -5 for DB2 and DB4 and changed from 50 to 20 with a step of -10 for DB1. The accuracy is viewed for the same training epoch. The efficiency of RCNN and CFF-RCNN is evaluated from three aspects: 1. The number of epochs for the same accuracy. 2. The training time for the same number of epochs. 3. The training time for the same accuracy. A 5-fold cross validation is used and classification accuracies on DB1, DB2 and DB4 are compared between RCNN and CFF-RCNN using Wilcoxon Matched-Pairs Signed-Ranks Test. The significance level is set to 0.05. We also tested the accuracy without using the k-fold cross validation, where the proportions of the training, testing and valid sets are 0.8, 0.1 and 0.1.

#### 3.3.2 Experiment 2: Robustness test

The second experiment is designed to test the performance of the networks when operating on a complex and chaotic dataset. Because sEMG signals vary significantly between subjects even with a precise electrode-placement control [52], the networks are usually trained specifically for each user in practice. In this experiment, both the RCNN and CFF-RCNN are trained using the total data of all the subjects in DB4 in order to test the robustness. The number of training epochs is 160.

## 4 Results

### 4.1 Performance evaluation

**Fig 5:**
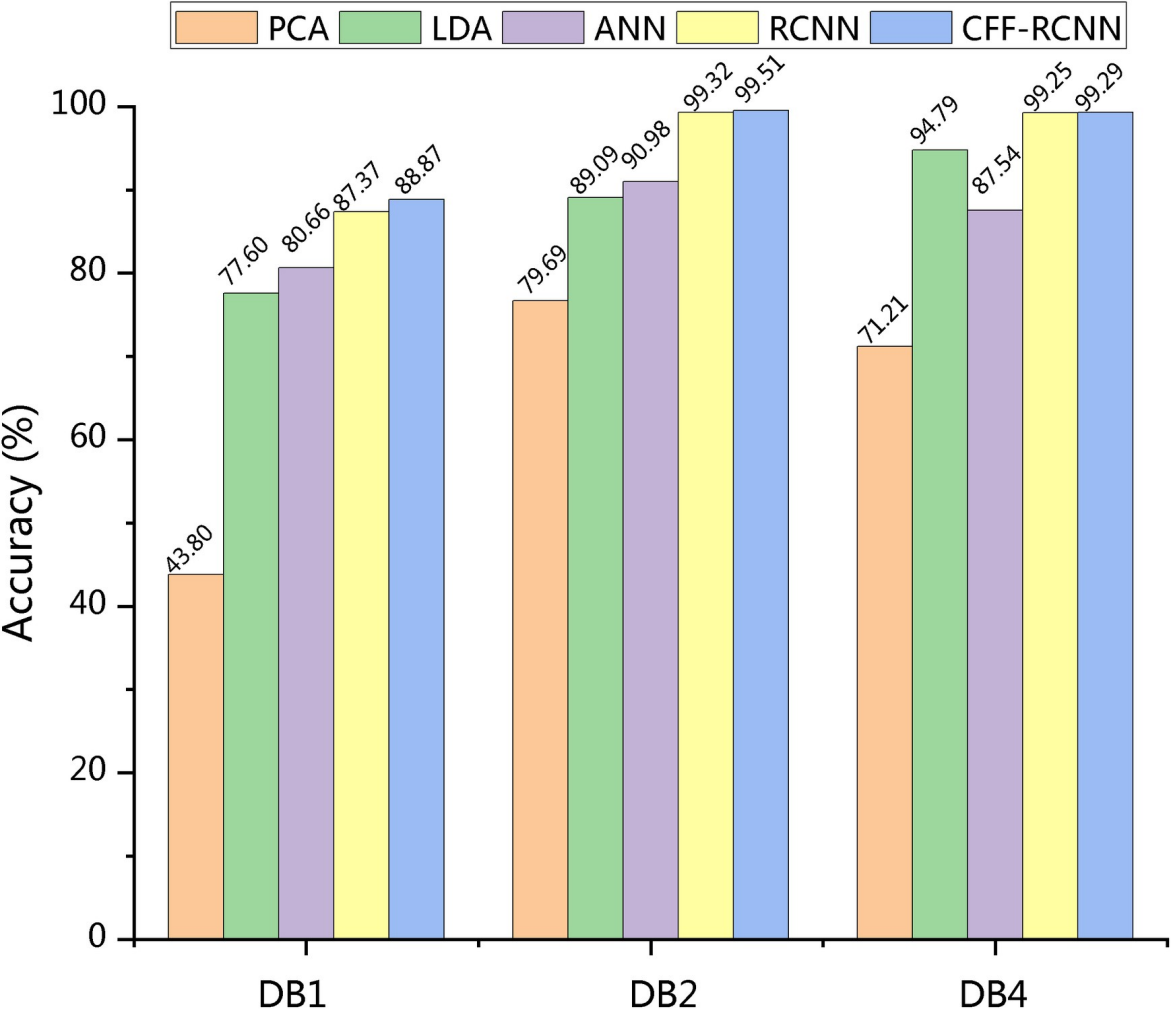
Classification accuracy of the proposed methods.

The classification accuracies of the machine learning methods, RCNN, and CFF-RCNN are illustrated in Fig 5. The accuracy in the figure is the mean value of all the subjects.

**Fig 6:**
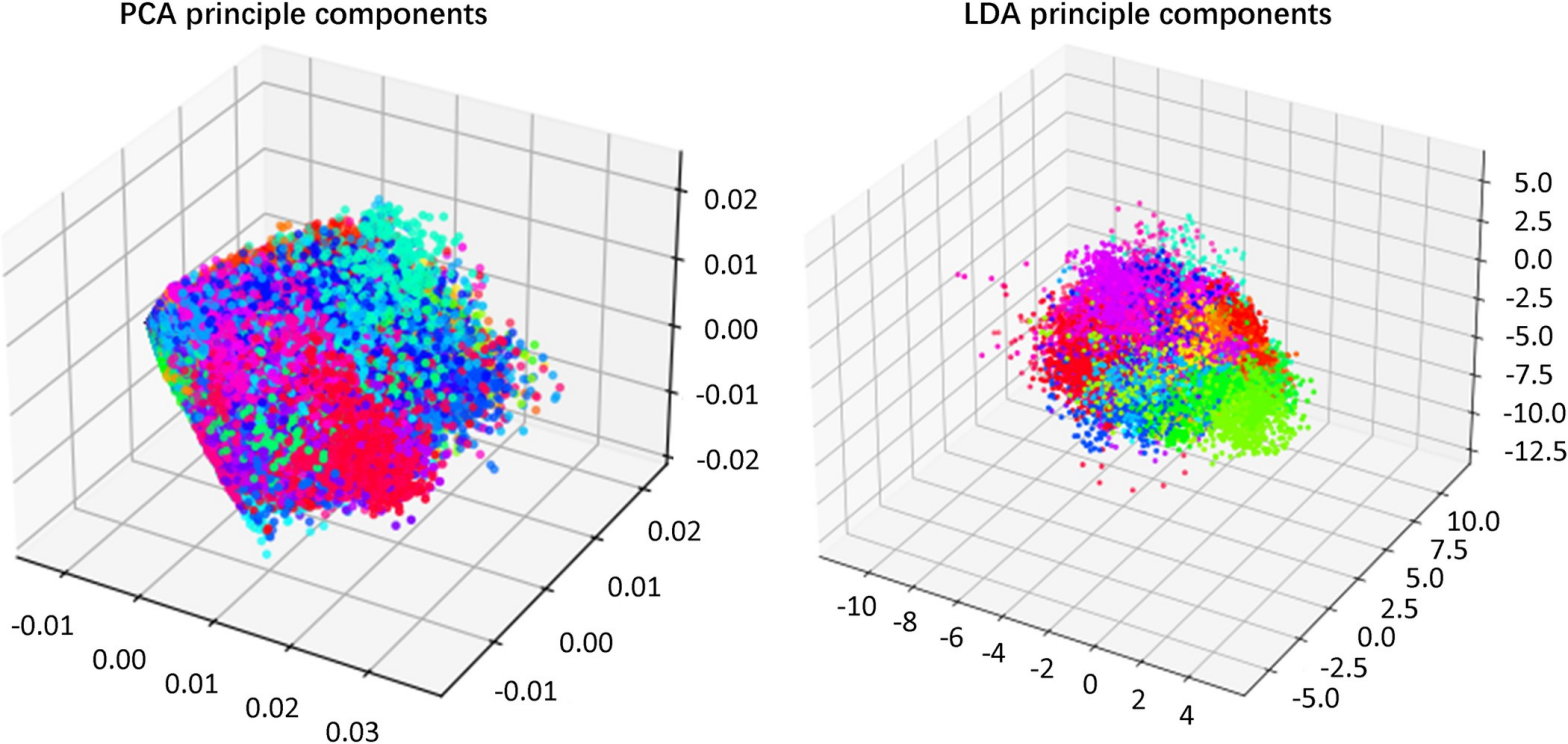
Principal components of DB1S1 obtained by the LDA and PCA.

Classification accuracies of the proposed methods are evaluated using DB1, DB2, and DB4 data. On DB1 data, accuracies in the machine learning models using the same data are 43.80%, 77.60%, and 80.66%, with the highest being 80.66% when ANN is applied. CFF-RCNN model reaches an accuracy of 88.87% and improves the accuracy by at least 1.50% compared to the RCNN. The top three principal components of Subject 1 in DB1, obtained by the PCA and LDA, are illustrated in Fig 6. Both of the clusters are hard to distinguish with groups of plots overlapping each other, meaning that the classifiers cannot find the differences among gestures efficiently; the LDA clusters have circular groupings and can be better represented by the cluster centroids, resulting in a higher classification accuracy of 77.60%. On DB2 data, the traditional RCNN achieves 99.32% of accuracy, which is 8.34% higher than that of the ANN methods. CFF-RCNN increases the accuracy of the RCNN from 99.32% to 99.51%. On DB4 data, the LDA performs the best among all the tested machine learning methods, with an accuracy of 94.79%. RCNN has a much higher accuracy of 99.25%. CFF-RCNN shows a slightly higher (99.29%) accuracy than RCNN.

Accuracy results of RCNN, CFF-RCNN and models from other works can be found in Table 3. When classifying hand gestures with more than 50 gestures, CFF-RCNN shows better performance compared with machine learnings and other state-of-the-arts.

**Table 3:** Classification accuracy of the compared methods.

Methods	DB1	DB2	DB4
LS-SVM (IAV+MAV+RMS+WL) [45]	85.19 ± 13.32%	-	-
LDA(IAV(or MAV)+ CC) [45]	84.23± 9.58%	-	-
RF [34, 44]	75.32%	75.27%	69.13 ± 7.77%
AtzoriNet [34]	-	60.3 ± 7.7%	-
ZhaiNet [46]	-	78.71%	-
TL-MKCNN [47]	-	86.67%	82.29%
FS-HGR [48]	-	85.94%	-
RNN with weight loss [49]	79.3%	78.0%	-
LSTM+MLP [50]	75.45±8.97%	-	-
CNN-LSTM [51]	-	79.329%	-
Attention-based hybrid CNN-RNN [32]	87%	82.2%	-
RCNN	87.37 ± 3.77%	99.32 ± 0.55%	99.25 ± 0.13%
CFF-RCNN	88.87 ± 3.63%	99.51 ± 0.12%	99.29 ± 0.10%

Based on these comparisons over the classification accuracies on DB1, DB2, and DB4 databases, the CFF strategy-integrated CFF-RCNN model outperforms traditional machine learning models and other state-of-the-arts, and further improves the classification result of a traditional RCNN architecture for all the tested datasets.

### 4.2 Comparison of the RCNN and CFF-RCNN

#### 4.2.1 Experiment 1: Accuracy and efficiency test

The prediction accuracies, total training times and training times per epoch are shown in Tables 4–6, corresponding to DB1, DB2 and DB4. The 5-fold cross validation is used for model comparison and the accuracy in the table is the mean value ± standard deviation of that of all the subjects.

**Table 4:** Predicting accuracy, total training time, and training time per epoch of the RCNN and CFF-RCNN on DB1 at different epochs, along with the statistical analyses results.

Number of epochs	DB1						
	Predicting Accuracy			Total training time		Training time per epoch	
	RCNN	CFF-RCNN	p-value	RCNN	CFF-RCNN	RCNN	CFF-RCNN
50	87.37 ± 3.77%	88.63 ± 3.66%	<0.05	2899.955s	2761.043s	57.999s	55.221s
40	86.33 ± 3.95%	87.92 ± 3.71%	<0.05	2353.686s	2245.716s	58.842s	56.143s
30	85.08 ± 4.06%	86.42± 4.01%	<0.05	1680.139s	1727.415s	56.005s	57.581s
20	82.85 ± 4.34%	84.23 ± 4.26%	<0.05	1210.431s	1097.884s	60.522s	54.894s

**Table 5:** Predicting accuracy, total training time, and training time per epoch of the RCNN and CFF-RCNN on DB2 at different epochs, along with the statistical analyses results.

Number of epochs	DB2						
	Predicting Accuracy			Total training time		Training time per epoch	
	RCNN	CFF-RCNN	p-value	RCNN	CFF-RCNN	RCNN	CFF-RCNN
30	99.32 ± 0.55%	99.51 ± 0.12%	<0.05	966.385s	1033.514s	32.213s	34.451s
25	99.15 ± 0.71%	99.44 ± 0.13%	<0.05	816.173s	910.904s	32.647s	36.436s
20	98.96 ± 0.67%	99.27 ± 0.20%	<0.05	698.677s	643.676s	34.934s	32.184s
15	98.06 ± 1.44%	98.80 ± 0.47%	<0.05	484.844s	542.245s	32.323s	36.150s
10	94.69 ± 2.61%	96.15 ± 1.51%	<0.05	323.722s	335.076s	32.372s	33.508s

**Table 6:** Predicting accuracy, total training time, and training time per epoch of the RCNN and CFF-RCNN on DB4 at different epochs, along with the statistical analyses results.

Number of epochs	DB4						
	Predicting Accuracy			Total training time		Training time per epoch	
	RCNN	CFF-RCNN	p-value	RCNN	CFF-RCNN	RCNN	CFF-RCNN
30	99.25 ± 0.13%	99.29 ± 0.10%	0.333	976.322s	1057.923s	32.544s	35.264s
25	99.12 ± 0.14%	99.23 ± 0.12%	<0.05	924.086s	950.205s	36.963s	38.008s
20	98.86 ± 0.19%	99.03 ± 0.22%	<0.05	731.771s	673.798s	36.589s	33.690s
15	97.88 ± 0.50%	98.30 ± 0.39%	<0.05	512.327s	576.734s	34.155s	38.449s
10	93.56 ± 1.49%	94.98 ± 1.34%	<0.05	378.690s	338.427s	37.869s	33.843s

The results shown in Tables 4–6 are analyzed by the accuracy and the efficiency (using epoch numbers and training times). We want to understand, when recognizing gestures with many classes, how the CFF strategy impacts the classification accuracy and the training time, and whether this new dimensionality reduction strategy is valid in feature extractions and improves the performance of the network.

First, to analyze accuracies, results are viewed for the same training epoch. Conclusions can be drawn that at each sampling point of the epochs, the CFF-RCNN has a higher classification accuracy than the RCNN on all the datasets. When the networks are trained on DB1, the accuracy of the CFF-RCNN is approximately 1–2% higher than that of the RCNN (see Table 4). For DB2 and DB4, the improvements, which are 1–2%, are more clear for smaller epochs (see Tables 5 and 6). Although the gap of accuracy between RCNN and CFF-RCNN decreases with a larger number of epochs, CFF-RCNN still achieves a higher accuracy result. This is likely because CFF-RCNN converges much faster than RCNN when moving close to their saturation stages.

The efficiency of RCNN and CFF-RCNN are evaluated using the epoch numbers. The CFF-RCNN achieves higher accuracy with fewer training epochs. For DB1, the RCNN reaches 86% of accuracy at 40 epochs and 87% of accuracy at 50 epochs, respectively. The CFF-RCNN reaches 86% of accuracy at 30 epochs and 87% of accuracy at 40 epochs, respectively. The CFF-RCNN experienced 10 epochs (20% of the total training epochs) fewer to reach the same level of accuracy as RCNN. To reach 99% of accuracy on DB2, CFF-RCNN uses 15 epochs, a 33% reduction in the number of epochs as compared to 25 epochs with RCNN. For DB4, the RCNN is trained with 20 and 30 epochs to achieve 98% and 99% of accuracy, respectively. The CFF-RCNN only uses 15 and 25 epochs, which is 5 epochs fewer for the same level of accuracy, corresponding to a reduction of 17% in the number of training epochs.

The efficiency of the CFF strategy is also assessed with the training times from the last four columns shown in Tables 4–6. Considering the training time for the same number of epochs, the CFF-RCNN sometimes has a slightly longer training time per epoch in general, increasing by 1–4 s, which is within tolerance (30 epochs in DB1; 30, 25, 15 and 10 epochs in DB2; 30, 25 and 15 epochs in DB4). At other epoch numbers, CFF-RCNN learns faster in a single epoch (50, 40 and 20 epochs in DB1; 20 epochs in DB2; 20 and 10 epochs in DB4). When trained on DB1, the CFF-RCNN has a better performance except at 30 epochs, illustrating that the CFF strategy can improve the feature extraction ability when the map size is small or the amount of data is limited.

The CFF-RCNN takes less time to train for the same accuracy. To achieve accuracies of 86%, 99% and 98% for DB1, DB2 and DB4, the CFF-RCNN uses 1727.415 s, 542.245 s and 576.734 s. The training time of the RCNN is 2353.686 s, 816.173 s and 731.771 s to arrive at the same accuracies. The CFF strategy reduces the training time by 26.61%, 33.56% and 21.19%, respectively.

**Fig 7:**
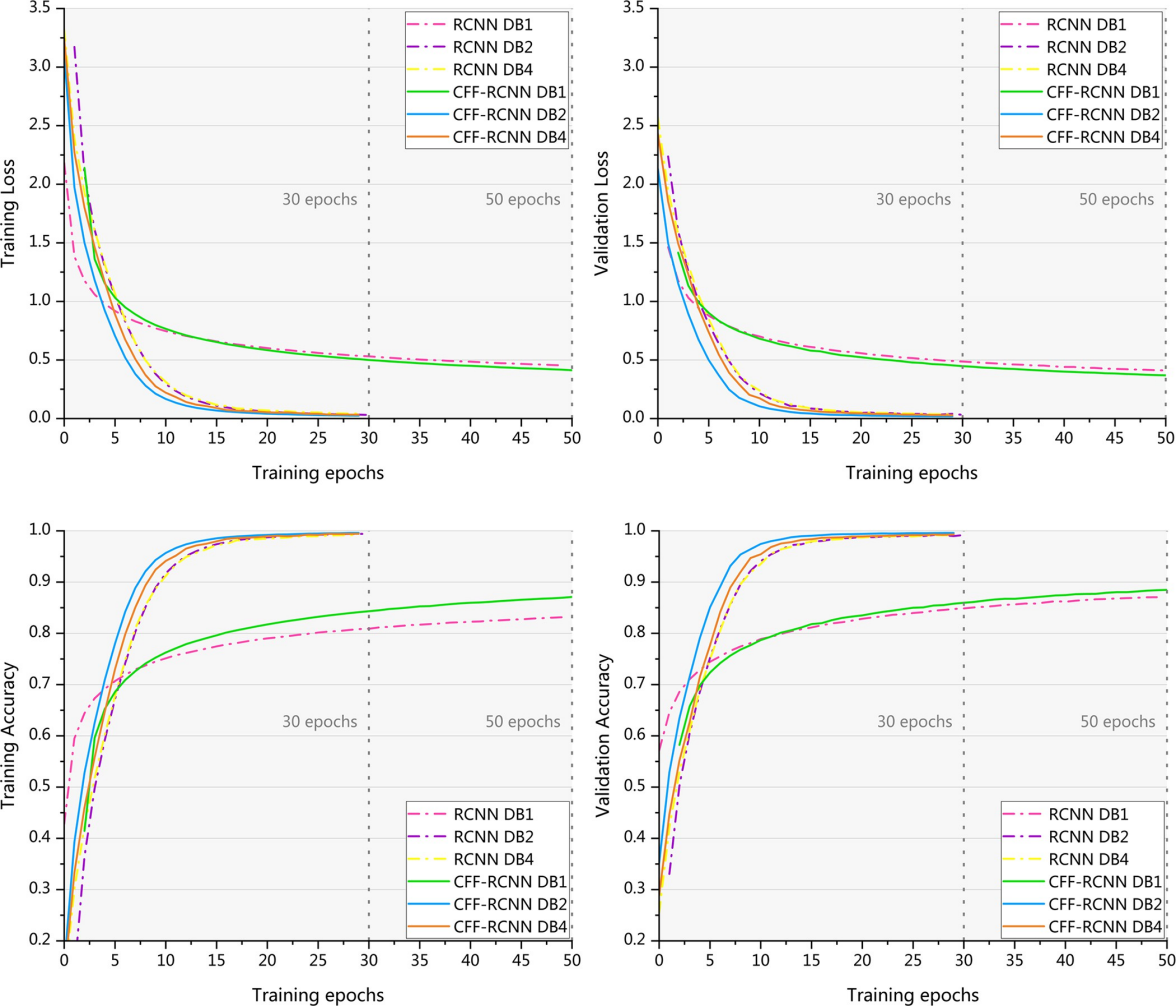
Loss and accuracy of the training and validation sets of the RCNN and CFF-RCNN on DB1, DB2 and DB4.

Fig 7 shows comparisons of the training loss, validation loss, training accuracy, and validation accuracy of RCNN and CFF-RCNN. The curves illustrate that the proposed CFF-RCNN has a smaller loss value, higher accuracy, and faster convergence speed than the RCNN.

**Fig 8:**
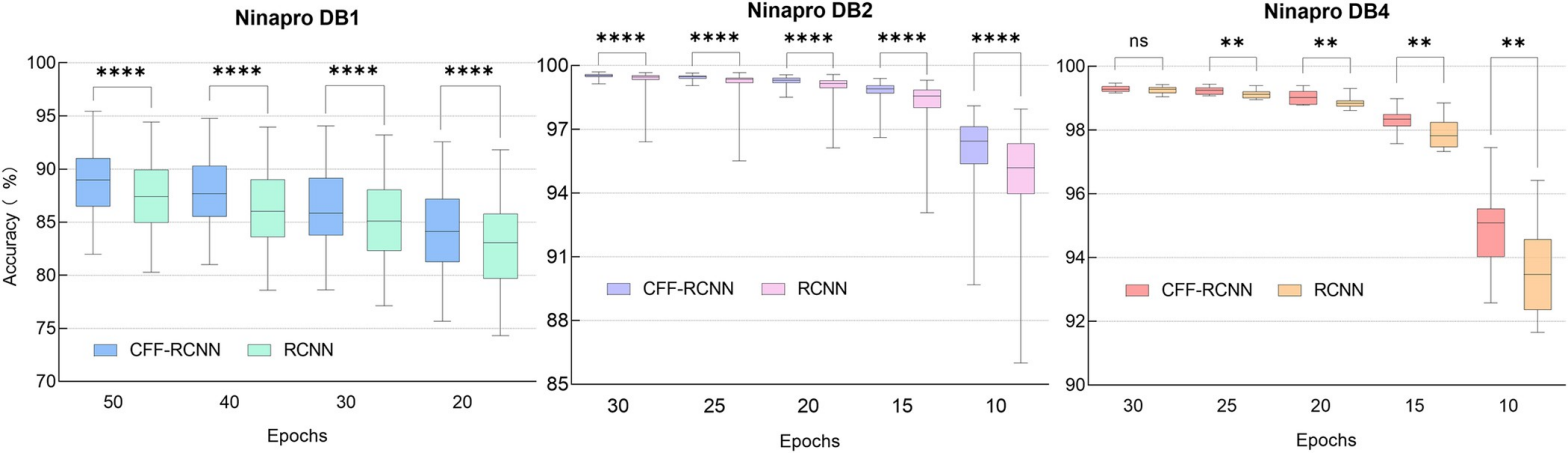
Comparisons between CFF-RCNN and RCNN on DB1, DB2 and DB4. P value style: 0.1234(ns), 0.0332(*), 0.0021(**),0.0002(***), <0.0001(****).

The statistical analysis results are illustrated in Tables 4–6 and Fig 8. Classification accuracies without using the k-fold cross validation can be found in S1–S3 Tables. Statistic analysis using the Wilcoxon Matched-Pairs Signed-Ranks Test revealed that CFF-RCNN significantly outperforms RCNN on DB1, DB2 and some sets of DB4 (p<0.05) (see Tables 4–6 and Fig 8). No significant difference (p>0.05) is found between the two methods when the number of epochs on DB4 is set to 30 (see Table 6 and Fig 8). Typically, when the number of epochs is smaller, the differences between RCNN and CFF-RCNN are more significant, indicating that CFF-RCNN has a better feature-extracting and learning ability with less training epochs.

#### 4.2.2 Experiment 2: Robustness test

**Fig 9:**
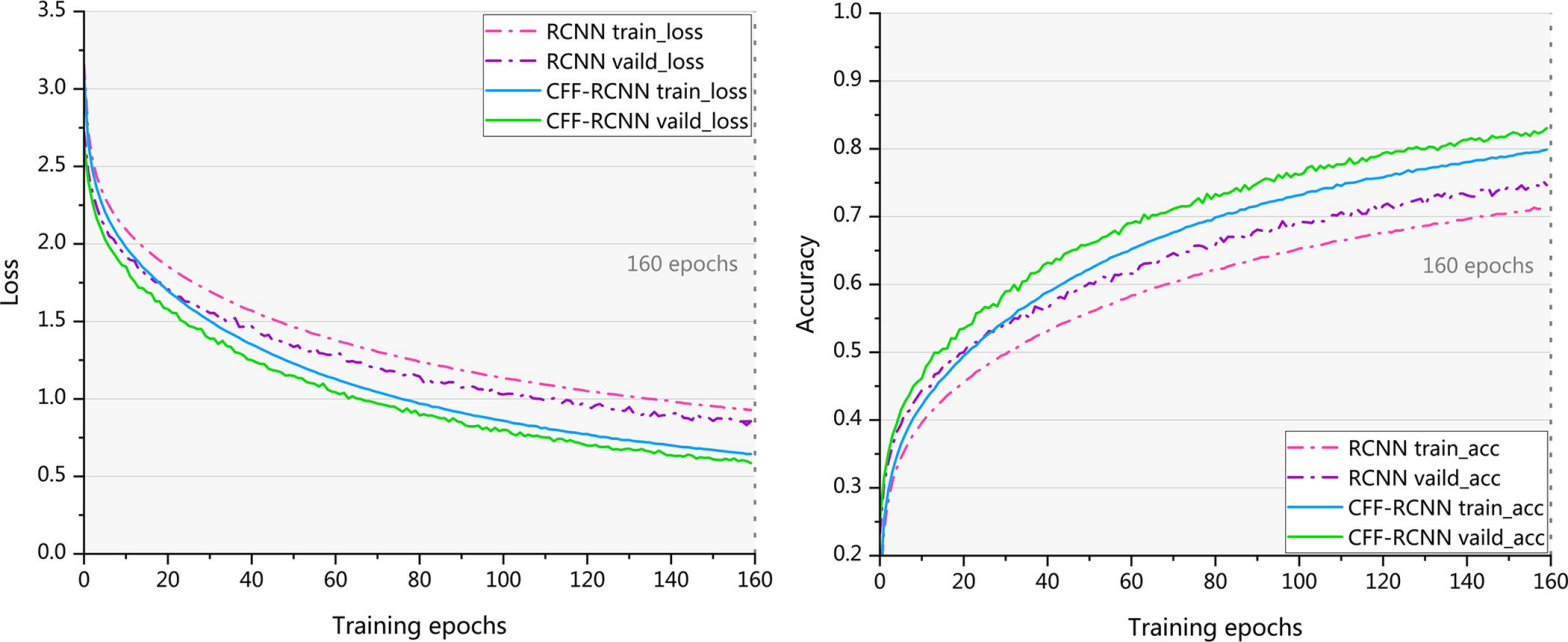
Loss and accuracy of the training and validation sets of the RCNN and CFF-RCNN on the total data of DB4.

As shown in Table 7, when trained using the total data of all the subjects in DB4, the classification accuracy of the CFF-RCNN in the testing set is 83.0495%, much higher than that of the RCNN (74.6528%). In the prediction set, RCNN has an accuracy of 74.18%, and CFF-RCNN has an accuracy of 83.1931%, which is 9% higher than its conventional counterpart. The training procedures are shown in Fig 9. In both loss and accuracy curves, the CFF-RCNN performs better in terms of a lower loss value, higher classification accuracy and faster convergence rate. The results indicate that the CFF-RCNN also shows a powerful data processing capability when dealing with mass and complicated datasets.

**Table 7:** Comparison of RCNN and CFF-RCNN on all the subjects in DB4.

DB4	RCNN	CFF-RCNN
	160 epochs	160 epochs
Training accuracy	71.3608%	79.8770%
Testing accuracy	74.6528%	83.0495%
Predicting accuracy	74.1787%	83.1931%

#### 4.2.3 Summary of the experiments

The CFF strategy is superior in two aspects. First, it helps to achieve a higher accuracy in gesture classification than the traditional RCNN from the training process to the end. Second, it has a faster convergence rate (1727.415 s, 542.245 s and 576.734 s to achieve accuracies of 86%, 99% and 98% for DB1, DB2 and DB4) compared to the RCNN (2353.686 s, 816.173 s and 731.771 s), and therefore shorten the training process (a reduction of 26.61%, 33.56% and 21.19% of the training time). These advantages play an important role in making the CFF-RCNN a qualified method of classifying hand gestures with more than 50 movements.

## 5 Discussion and conclusions

In this article, we proposed and applied CFF strategy as a new dimensionality reduction strategy in an improved CFF-RCNN structure for sEMG-based hand gesture recognition tasks with more than 50 classes. The CFF strategy concatenates a pooling layer and a convolutional layer with a stride of 2, which reduces the information-loss that normally arises from models carrying a single pooling or convolutional layer.

Evaluations have been made thoroughly using three benchmark databases in the NinaPro database: DB1, DB2 and DB4. We compared the classification accuracies of the proposed CFF-RCNN model with traditional machine learnings and other state-of-the-art methods. Results showed that the CFF-RCNN achieved better accuracies of 88.87% in DB1 (53 gestures), 99.51% in DB2 (50 gestures) and 99.29% in DB4 (53 gestures). To demonstrate that the CFF strategy can also improve efficiency, we further designed the training process test and the complex dataset tolerance test to compare the RCNN and CFF-RCNN, and found that CFF-RCNN improved the convergence rate by 26.61%, 33.56% and 21.19%, which significantly shortened the required training time.

The present study is aimed at optimizing the network architecture for a better performance of feature-extracting in experiment settings. However, there are three main limitations of the present work. First, all the experiments are based on the NinaPro database. To explore potential clinical application of the CFF-RCNN model, accuracy and convergence rate can be tested using clinical data. Second, the present network is limited to offline training and testing, whereas in clinic applications such as prosthesis operation and stroke rehabilitation. Third, the parameters of the network were trained specifically for each user. Deep learning requires an unreasonable amount of effort from a single user in order to generate tens of thousands of examples as training data [53]. Therefore, new technologies such as transfer learning (TL) may be considered in order to lighten the user’s workload [53].

In the future, more works are required to overcome the drawbacks mentioned above. We can test the performance of the CFF-RCNN using data recorded by our sensing system. Experiments can be done on both intact and amputated subjects. The structure of the proposed model will be further implemented locally on wearable devices to achieve real-time classification. To reduce the training effort of a single person, new technologies, such as TL and other state-of-the-art scheme, can be added to the present structure as well. With these future improvements, the CFF strategy together with the CFF-RCNN model could enable a wider range of applications requiring multi-class recognitions, real-time classifications, and low-workload training demands.

## Supporting information

S1 Table Predicting accuracy without k-fold cross validation on DB1. (DOCX)

S2 Table Predicting accuracy without k-fold cross validation on DB2. (DOCX)

S3 Table Predicting accuracy without k-fold cross validation on DB4. (DOCX)
