# Supplementary material for: A novel concatenate feature fusion RCNN architecture for sEMG-based hand gesture recognition
Source: PLoS One. 2022 Jan 20;17(1):e0262810. doi: 10.1371/journal.pone.0262810 (PMC8775254; doi:10.1371/journal.pone.0262810)
Supplement: S1 Table — (DOCX) [file pone.0262810.s001.docx]

**S1 Table. Predicting accuracy without k-fold cross validation on DB1.**

| Number of epochs | DB1 | | |
| --- | --- | --- | --- |
|  | Predicting Accuracy | | |
|  | RCNN | CFF-RCNN | p-value |
| 50 | 87.16 ± 3.72% | 88.63 ± 3.66% | <0.05 |
| 40 | 86.24 ± 3.97% | 87.64 ± 3.86% | <0.05 |
| 30 | 84.71 ± 4.20% | 86.17 ± 4.04% | <0.05 |
| 20 | 82.68 ± 4.25% | 83.98 ± 4.28% | <0.05 |
