# Supplementary material for: A novel concatenate feature fusion RCNN architecture for sEMG-based hand gesture recognition
Source: PLoS One. 2022 Jan 20;17(1):e0262810. doi: 10.1371/journal.pone.0262810 (PMC8775254; doi:10.1371/journal.pone.0262810)
Supplement: S2 Table — (DOCX) [file pone.0262810.s002.docx]

**S2 Table. Predicting accuracy without k-fold cross validation on DB2.**

| Number of epochs | DB2 | | |
| --- | --- | --- | --- |
|  | Predicting Accuracy | | |
|  | RCNN | CFF-RCNN | p-value |
| 30 | 99.26 ± 1.34% | 99.61 ± 1.32% | <0.05 |
| 25 | 99.06 ± 1.55% | 99.54 ± 0.18% | <0.05 |
| 20 | 98.92 ± 1.28% | 99.38 ± 0.25% | <0.05 |
| 15 | 98.09 ± 2.61% | 99.01 ± 0.45% | <0.05 |
| 10 | 94.43 ± 3.60% | 96.51 ± 1.90% | <0.05 |
