# Supplementary material for: A novel concatenate feature fusion RCNN architecture for sEMG-based hand gesture recognition
Source: PLoS One. 2022 Jan 20;17(1):e0262810. doi: 10.1371/journal.pone.0262810 (PMC8775254; doi:10.1371/journal.pone.0262810)
Supplement: S3 Table — (DOCX) [file pone.0262810.s003.docx]

**S3 Table.** **Predicting accuracy without k-fold cross validation on DB4.**

| Number of epochs | DB4 | | |
| --- | --- | --- | --- |
|  | Predicting Accuracy | | |
|  | RCNN | CFF-RCNN | p-value |
| 30 | 99.17 ± 0.16% | 99.22 ± 0.21% | 0.739 |
| 25 | 98.99 ± 0.26% | 99.14 ± 0.12% | <0.05 |
| 20 | 98.5915% | 98.88% | <0.05 |
| 15 | 97.76 ± 0.43% | 98.26 ± 0.55% | 0.058 |
| 10 | 92.57 ±2.02% | 94.98 ± 1.55% | <0.05 |
